# Sirt6 overexpression suppresses senescence and apoptosis of nucleus pulposus cells by inducing autophagy in a model of intervertebral disc degeneration

**DOI:** 10.1038/s41419-017-0085-5

**Published:** 2018-01-19

**Authors:** Jian Chen, Jun-Jun Xie, Meng-Yun Jin, Yun-Tao Gu, Cong-Cong Wu, Wei-Jun Guo, Ying-Zhao Yan, Zeng-Jie Zhang, Jian-Le Wang, Xiao-Lei Zhang, Yan Lin, Jia-Li Sun, Guang-Hui Zhu, Xiang-Yang Wang, Yao-Sen Wu

**Affiliations:** 1 Department of Orthopaedic Surgery, The Second Affiliated Hospital and Yuying Children’s Hospital of Wenzhou Medical University, Wenzhou, 325027 People’s Republic of China; 2 School of Pharmaceutical Sciences, Key Laboratory of Biotechnology and Pharmaceutical Engineering, Wenzhou Medical University, Wenzhou, 325027 People’s Republic of China; 3 The Second Affiliated Hospital and Yuying Children’s Hospital of Wenzhou Medical University, Wenzhou, 325027 People’s Republic of China

## Abstract

Treatment of intervertebral disc degeneration (IDD) seeks to prevent senescence and death of nucleus pulposus (NP) cells. Previous studies have shown that sirt6 exerts potent anti-senescent and anti-apoptotic effects in models of age-related degenerative disease. However, it is not known whether sirt6 protects against IDD. Here, we explored whether sirt6 influenced IDD. The sirt6 level was reduced in senescent human NP cells. Sirt6 overexpression protected against apoptosis and both replicative and stress-induced premature senescence. Sirt6 also activated NP cell autophagy both in vivo and in vitro. 3-methyladenine (3-MA) and chloroquine (CQ)-mediated inhibition of autophagy partially reversed the anti-senescent and anti-apoptotic effects of sirt6, which regulated the expression of degeneration-associated proteins. In vivo, sirt6 overexpression attenuated IDD. Together, the data showed that sirt6 attenuated cell senescence, and reduced apoptosis, by triggering autophagy that ultimately ameliorated IDD. Thus, sirt6 may be a novel therapeutic target for IDD treatment.

## Introduction

Intervertebral disc degeneration (IDD) is an age-related degenerative disease and the major cause of low back pain, reducing quality-of-life and creating a large economic burden^1^. Various patient-specific and external factors, including age, a genetic predisposition, and mechanical stress, contribute to IDD initiation and progression^2–4^. However, the specific molecular mechanism of disease development has not been elucidated and no effective treatment is available.

The intervertebral disc contains three integrated structures: the gelatinous inner nucleus pulposus (NP), the outer annulus fibrosus, and cartilaginous endplates, facilitating mechanical spinal function. IDD is an abnormal cell-mediated process culminating in structural failure^5,6^. Cells of the NP control extracellular matrix (ECM) metabolism and play a critical role in IDD^7^. With aging and degeneration, the number of NP cells decreases, which is attributable to both cell death and senescence^8^. Moreover, increasing evidence shows that prevention of apoptosis and senescence may ameliorate IDD induced by factors such as interleukin (IL)-1β and reactive oxygen species (ROS). Therefore, studies on NP cell senescence and apoptosis may allow IDD pathogenesis to be better understood, and may identify new therapeutic targets.

The sirtuins are a family of NAD^+^-dependent histone deacetylases (sirt 1–7), which protect against age-related diseases, including cancer, neurodegeneration, and cardiovascular conditions^9,10^. Sirt6-transgenic mice live longer than wild-type animals^11^, and sirt6 −/− mice exhibit degenerative and metabolic defects reminiscent of premature aging. In hepatocellular carcinoma cells, sirt6 deacetylates Ku70, blocking the binding thereof to Bax and reducing apoptosis^12^. However, to the best of our knowledge, no study has yet sought a relationship between sirt6 status, senescence, and apoptosis during IDD, either in vitro or in vivo.

In the present work, we showed that sirt6 overexpression ameliorated IDD progression by inhibiting NP cell senescence and stress-induced apoptosis. In vitro, sirt6 countered IL-1β-induced NP cell senescence and apoptosis, as revealed using a rat annulus needle puncture model.

## Results

### Sirt6 level declines in senescent NP cells both in vivo and in vitro

**Fig. 1:**
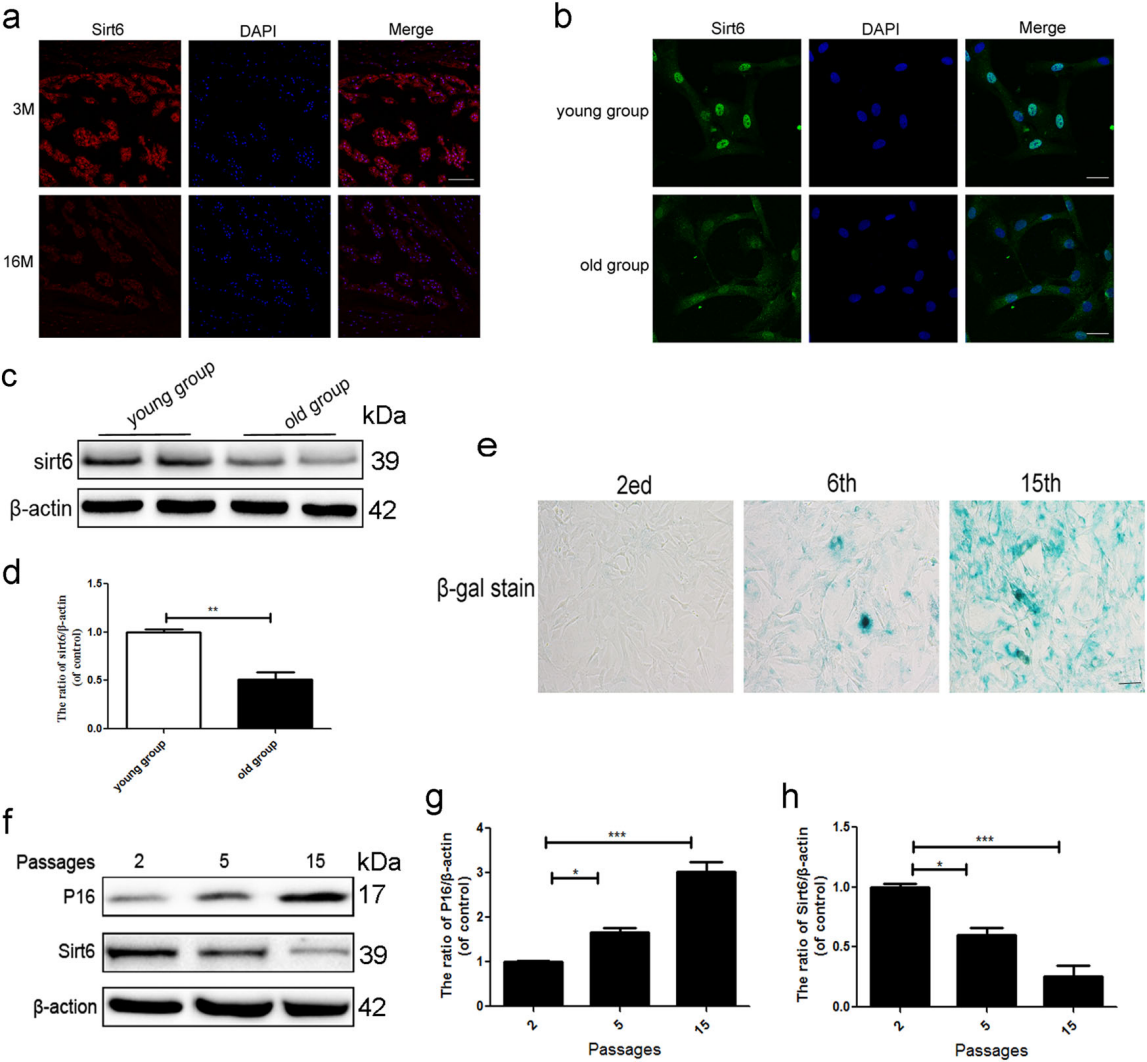
Sirt6 level declines in senescent NP cells both in vivo and in vitro **a** Immunofluorescence of sirt6 in aging (16 months) group and young (3 months) group (scale bar: 200 μm). **b** Immunofluorescence of sirt6 in NP cells that were isolated from aging and young rats (scale bar: 50 μm). **c**,** d** Representative western blots and quantification data of sirt6 in NP cells of each group. **e** SA-β-gal staining assay was performed in rat NP cells as treated above (scale bar: 50 μm). **f**–**h** Representative western blots and quantification data of p16 and sirt6 in NP cells of each group. Columns represent mean ± SD. Significant differences between the treatment and control groups are indicated as *P < 0.05, **P < 0.01, ***P < 0.001, *n* = 5

IDD is strictly age-dependent, and the sirt6 level declines with aging in many tissues^13^. Fig. 1a shows that sirt6 fluorescence intensity in the NP area was lower in old rats (16 months) compared with young rats (3 months). Our immunofluorescence and western blotting data showed that the sirt6 level was lower in second-passage NP cells from old patients than young patients (Fig. 1b and c). We also measured sirt6 levels at passages 2, 5, and 15 and evaluated senescence by SA-β-gal activity and p16 (specific indicators of senescence) level. The SA-β-gal levels increased as the number of passages rose (Fig. 1e). Furthermore, sirt6 was downregulated and p16 upregulated in primary NP cultures of rat as the number of passages increased (replicative senescence) (Fig. 1f–h). Thus, the Sirt6 level fell as cells entered senescence.

### Sirt6 overexpression attenuated replicative senescence of rat NP cells

**Fig. 2:**
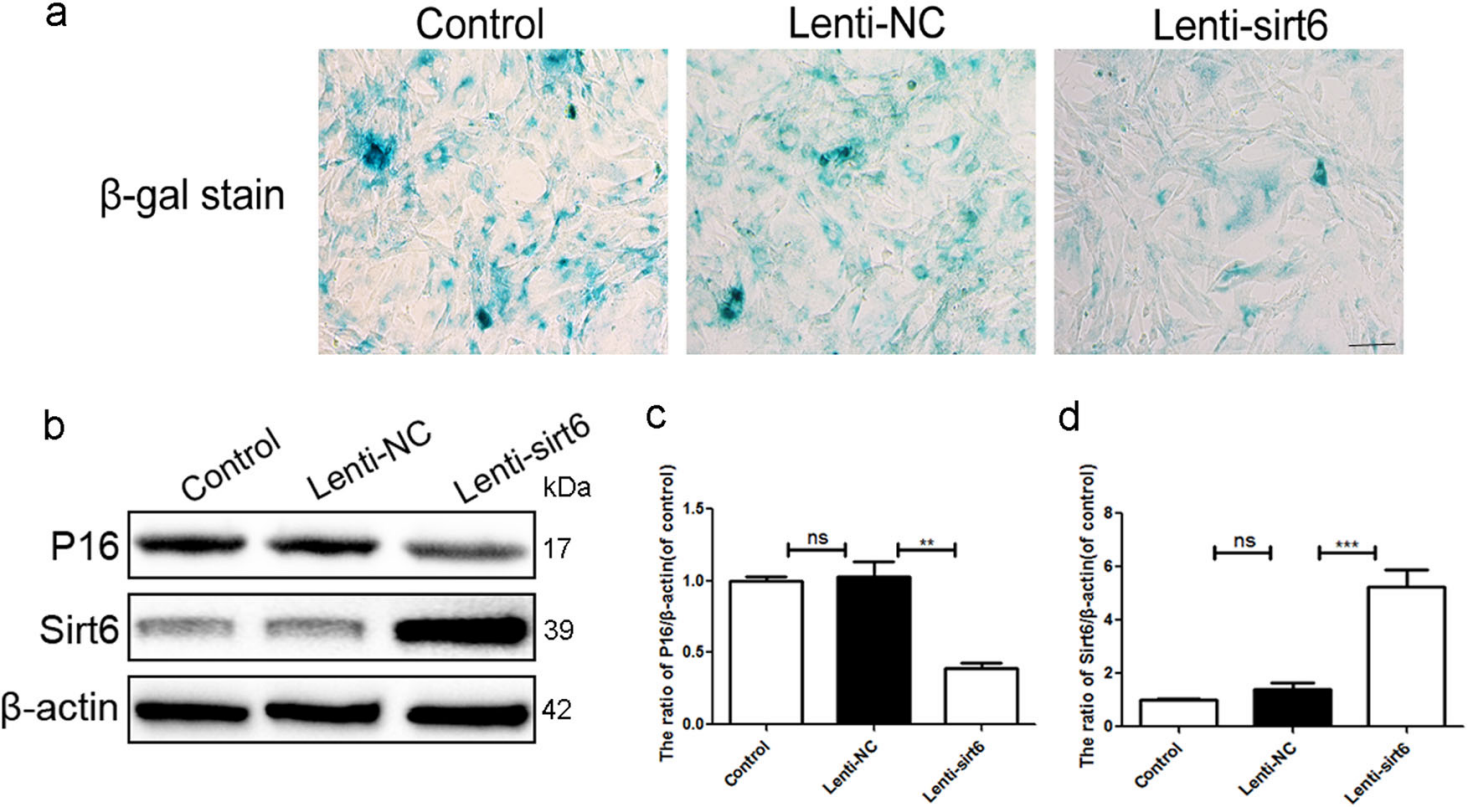
Sirt6 overexpression attenuated replicative senescence of rat NP cells We passaged NP cells to the 15th generations to detect sirt6 and p16. **a** SA-β-gal staining assay was performed in NP cells of each group (scale bar: 50 μm). **b**–**d** Representative western blots and quantification data of p16 and sirt6 in human NP cells of each group; columns represent mean ± SD. Significant differences between the treatment and sham groups are indicated as **P < 0.01, ***P < 0.001, *n* = 5

We found that sirt6 expression decreased in senescent NP cells. We thus transfected NP cells with Lenti-sirt6 to overexpress the sirt6 protein. At passage 15, these cells exhibited less SA-β-gal activity than control cells (Fig. 2a). p16 expression was also reduced (Fig. 2b–d), suggesting that sirt6 played a critical role in NP cell senescence.

### Sirt6 overexpression suppressed IL-1β-induced premature senescence and apoptosis of human NP cells

**Fig. 3:**
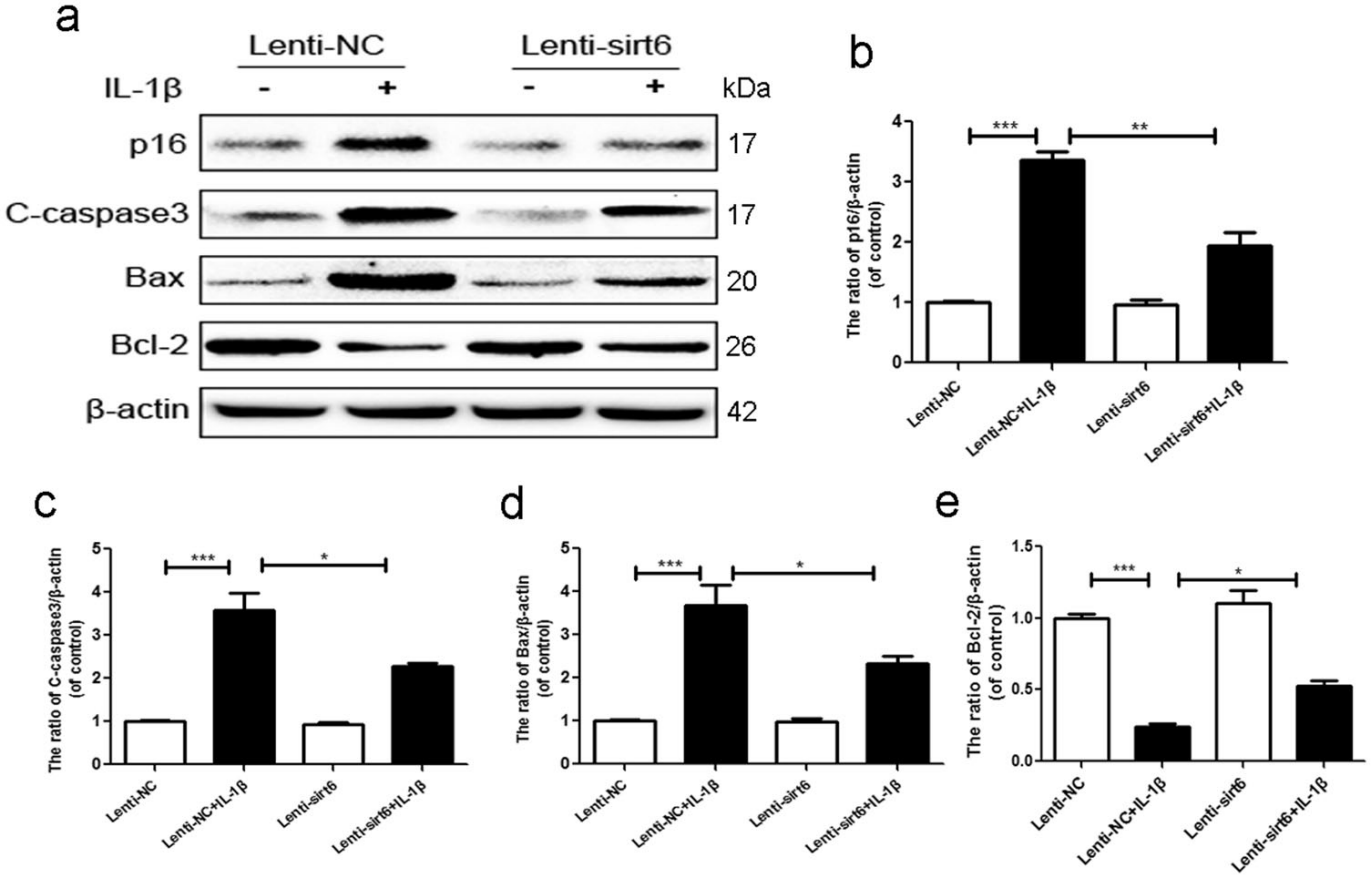
Sirt6 overexpression suppressed IL-1β-induced premature senescence and apoptosis of human NP cells **a**–**e** Representative western blots and quantification data of p16, cleaved caspase3, Bax, Bcl-2 in NP cells of each group as treated above; columns represent mean ± SD. Significant differences between the treatment and control groups are indicated as *P < 0.05, **P < 0.01, ***P < 0.001, *n* = 5

IDD involves both age-related and stress-induced tissue damage; cell senescence may be accelerated by stressful events^8^. Figure 3a shows that IL-1β significantly increased the NP cell level of p16, indicating that IL-1β induced premature senescence, and that this was inhibited by sirt6. We also measured the levels of Bcl-2, Bax, and cleaved caspase 3 (markers of apoptosis). Bcl-2 inhibits apoptosis, Bax is released upon induction of apoptosis, and cleaved caspase 3 mediates the cleavage of various cellular components^14,15^. Compared with the Lenti-NC group, IL-1β greatly upregulated the levels of Bax and cleaved caspase 3, but these increases were significantly lower in the Lenti-sirt6 group. Sirt6 overexpression upregulated production of the anti-apoptotic protein Bcl-2 (Fig. 3). Thus, sirt6 overexpression inhibited IL-1β-induced apoptosis.

### Sirt6 overexpression activated human NP cell autophagy

**Fig. 4:**
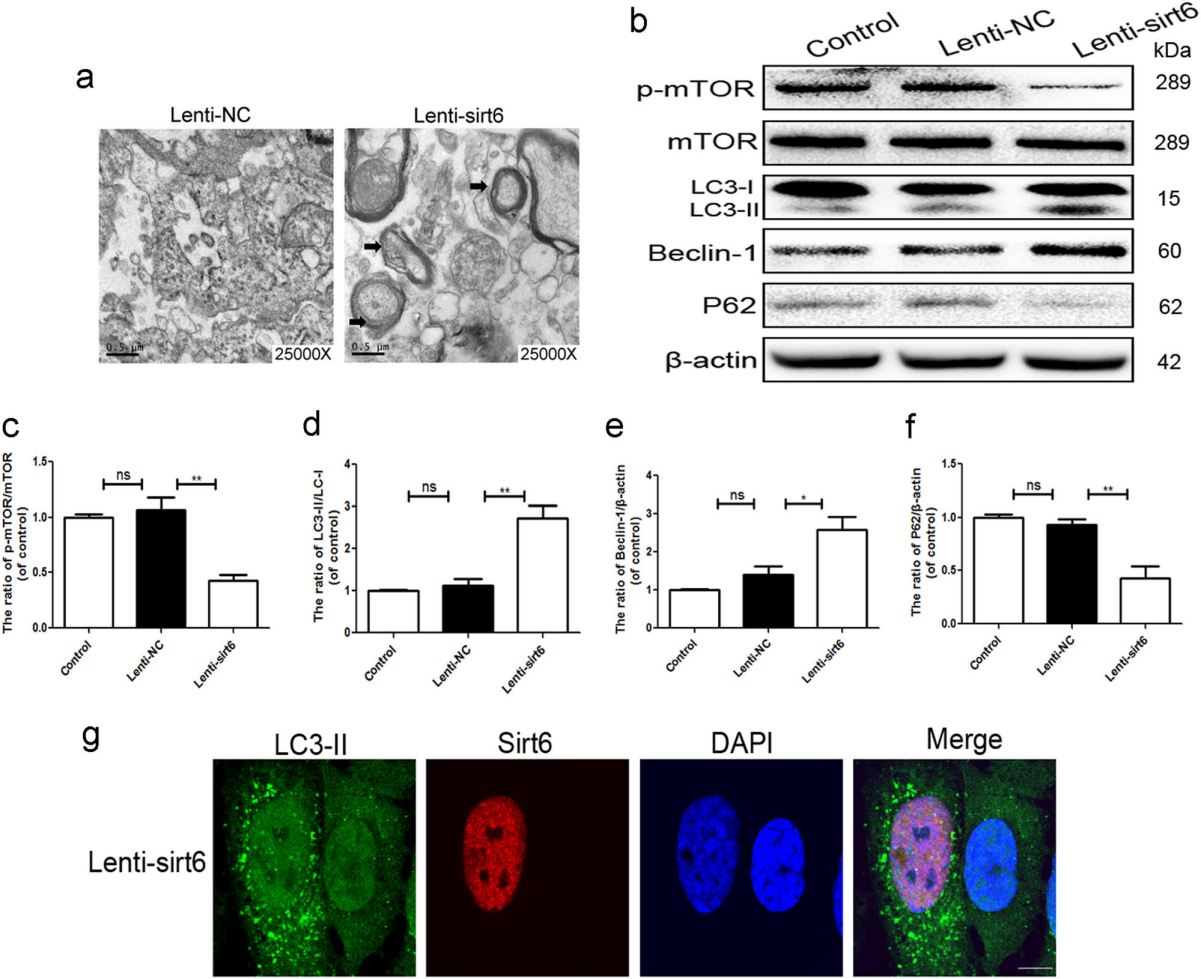
Sirt6 overexpression activated human NP cell autophagy **a** Transmission electron microscopy showed the autophagosomes (black arrow: autophagosome) in NP cells after lentivirus transfection. **b**–**f** Representative western blots and quantification data of p-mTOR, mTOR, LC3, P62, and Beclin-1 protein in NP cells of each group; columns represent mean ± SD. Significant differences between the treatment and control groups are indicated as **P < 0.01, *P < 0.05, *n* = 5. **g** Double immunofluorescence of sirt6 (red) and LC3-II (green) in NP cells treated by Lenti-sirt6 (scale bar: 10 μm)

TEM revealed that the numbers of neuronal autophagosomal vacuoles increased after sirt6 transfection, indicating that autophagy was in play (Fig. 4a). The LC3-II/LC3-I ratio, and the Beclin-1 and P62 levels (markers of autophagy), were tested via western blot assay. The LC3-II/LC3-I ratio and the level of Beclin-1 were higher in the Lenti-sirt6 than the Lenti-NC group, but the level of p62 was lower in the former group (Fig. 4b–f), consistent with the immunofluorescence data. The Lenti-sirt6 group showed more accumulation of LC3-positive puncta, which was the marker of autophagic vacuoles, compared to Lenti-NC group (Supplementary Figure S2a–c). Meanwhile, to further confirm this phenomenon, we used low titer of lenti-sirt6 virus to transfect cells, leading to uneven transfection distribution (Supplementary Figure S2d). As shown in Fig. 4g, we noted that the cell that was transfected by lenti-sirt6 had more autophagic vacuoles. However, the cell that was not transfected by lenti-sirt6 showed lower autophagic vacuoles.

mTOR is a critical regulator of autophagy, which was induced by a series of molecular events, such as deletion of biological factors, decrease of intracellular energy, or amino acid availability, and then transmitted to downstream effectors^16^. The activity of mTOR was measured via testing the phosphorylation of mTOR. As shown in Fig. 4b and c, sirt6 overexpression inhibited the phosphorylation of mTOR, suggesting that sirt6 regulated the autophagy via inhibiting the mTOR signaling.

### Inhibition of autophagy attenuated the anti-apoptotic effect of sirt6

**Fig. 5:**
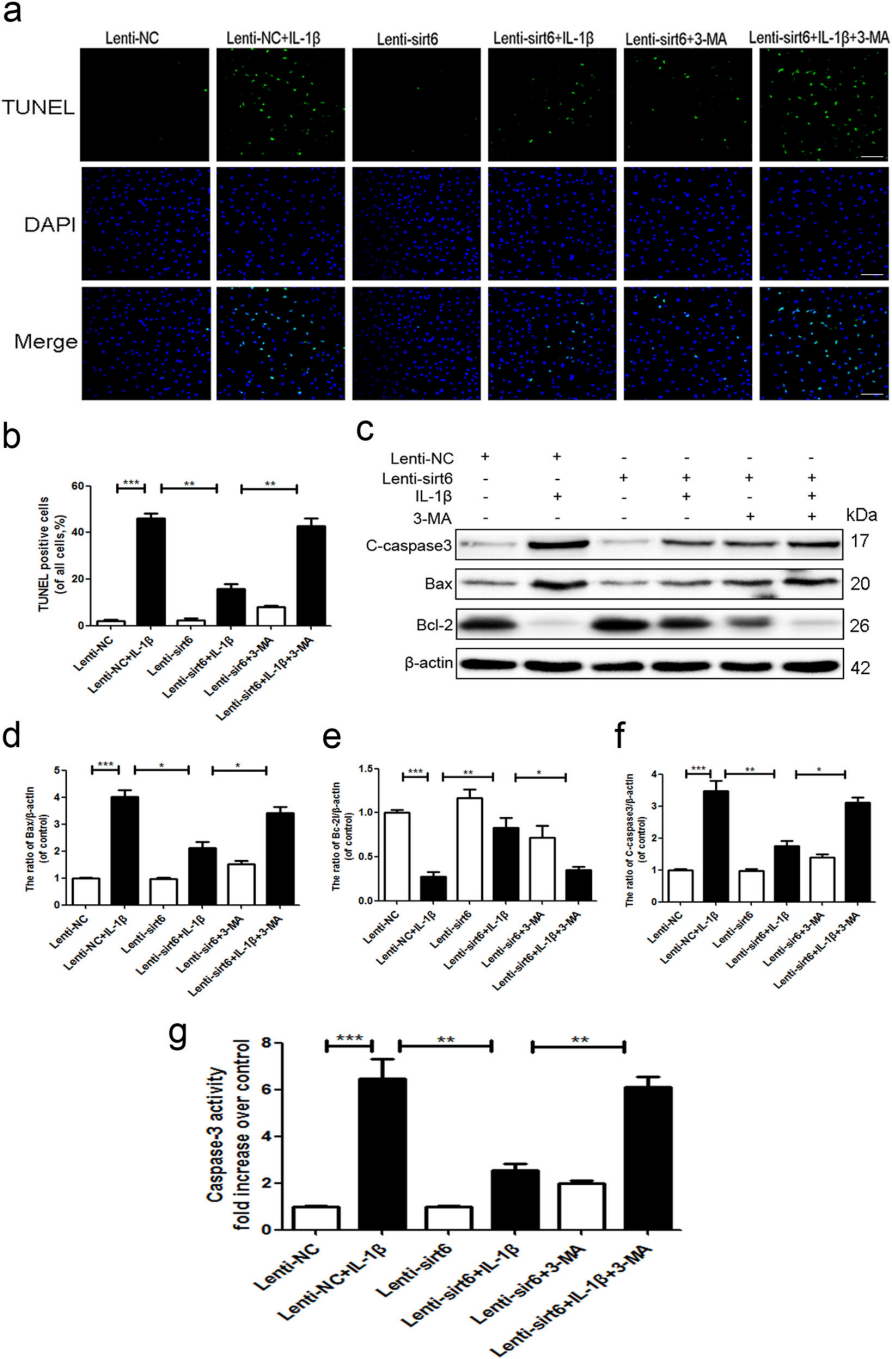
Inhibition of autophagy attenuated the anti-apoptotic effect of sirt6 **a**,** b** TUNEL assay was performed to assess the apoptosis in NP cells of each group as treated above (scale bar: 50 μm). **c**–**f** Representative western blots and quantification data of cleaved caspase3, Bax, Bcl-2 in NP cells of each group as treated above. **g** Caspase3 activity in NP cells of each group. Columns represent mean ± SD. Significant differences between the treatment and control groups are indicated as *P < 0.05, *n* = 5

To determine whether autophagy was related to the anti-apoptotic effect of sirt6, NP cells were pretreated with the classical autophagy inhibitor 3-methyladenine (3-MA), followed by TUNEL staining. Compared with the Lenti-NC group, the Lenti-NC + IL-1β group exhibited a greater number of apoptotic cells. However, sirt6 overexpression greatly reduced apoptotic activity, but this effect was reversed by 3-MA (Fig. 5a and b). Western blotting showed that the IL-1β-mediated upregulation of cleaved caspase 3 and Bax was significantly attenuated by sirt6 overexpression. Combined sirt6 transfection and 3-MA treatment increased the expression levels of both cleaved caspase 3 and Bax, and 3-MA significantly reduced the level of Bcl-2 (Fig. 5c–f). Caspase-3 activity assay showed that sirt6 overexpression inhibited the IL-1β-induced activation of caspase-3 activity, which was reversed by 3-MA (Fig. 5g). To further identify whether autophagy was involved in the anti-apoptotic effects of sirt6, chloroquine (CQ), another autophagylysosome pathway inhibitor, which inhibits autophagosomal and lysosomal fusion, was used to treated with Lenti-sirt6. Similarly, CQ abolished the anti-apoptotic sirt6 (Supplementary Figure S2a and b). Together, the results show that autophagy is essential when sirt6 increases survival.

### Sirt6 inhibits stress-induced premature senescence via autophagy

**Fig. 6:**
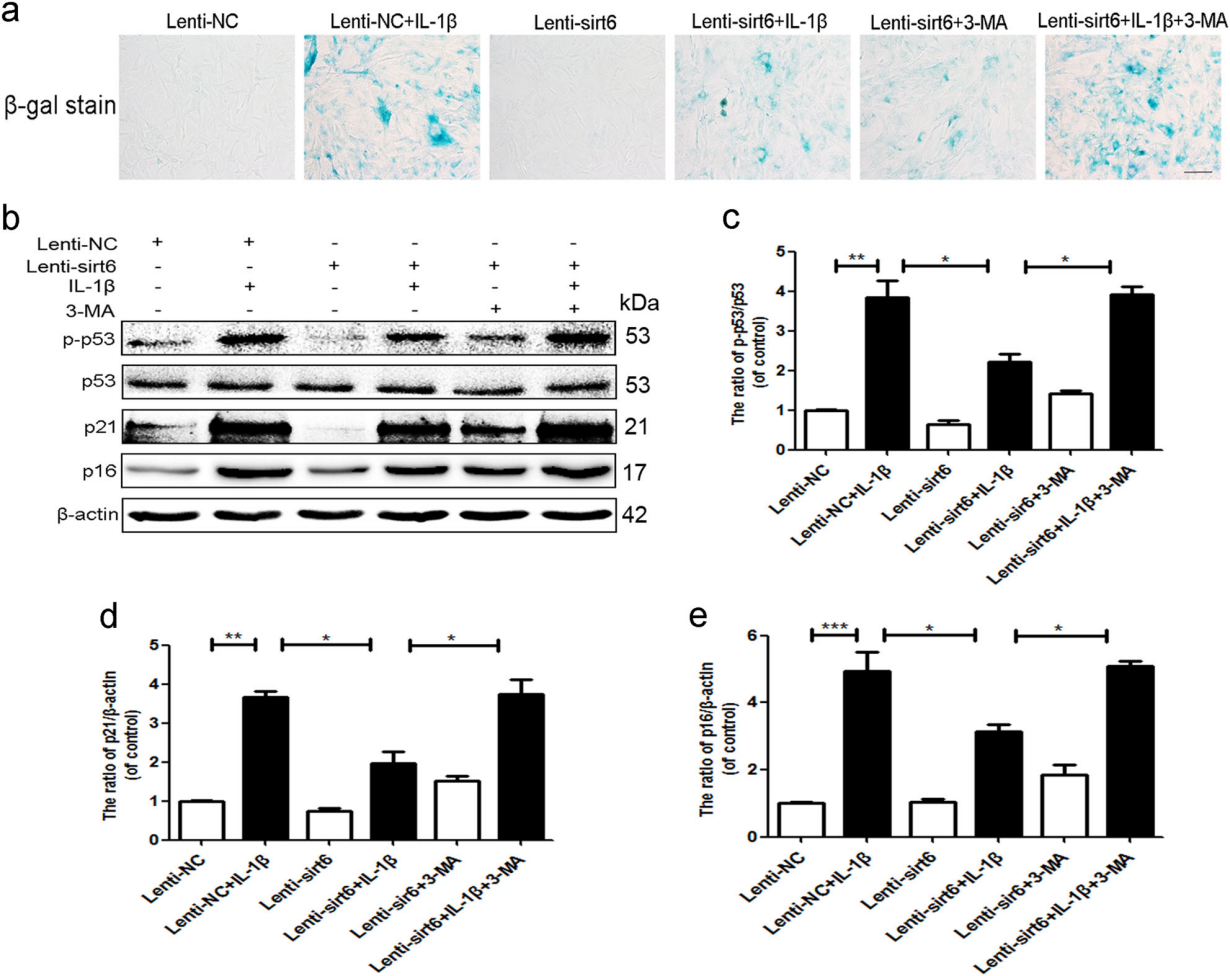
Sirt6 inhibits stress-induced premature senescence via autophagy **a** SA-β-gal staining assay was performed in NP cells of each group as treated above (scale bar: 50 μm). **b**–**e** Representative western blots and quantification data of p-p53, p53, p21, and p16 in NP cells of each group as treated above; columns represent mean ± SD. Significant differences between the treatment and control groups are indicated as *P < 0.05, **P < 0.01, ***P < 0.001, *n* = 5

The cyclin-dependent kinase inhibitors, such as p16, p21, and p53, were canonical markers of cellular senescence^17,18^. As shown in Fig. 6, IL-1β significantly upregulated SA-β-gal activity and the radio of p-p53/p53, p21, p16 levels, whereas sirt6 transfection inhibited these effects. However, inhibition of autophagy with 3-MA abolished the anti-senescence effects of sirt6. Meanwhile, inhibition of autophagy with CQ reversed the anti-senescence effects of sirt6 (Supplementary Figure S2c), further indicating that sirt6 played a critical role in regulation of stress-induced premature senescence via autophagy.

### Sirt6 regulated the expression levels of degeneration-associated proteins via autophagy of human NP cells

**Fig. 7:**
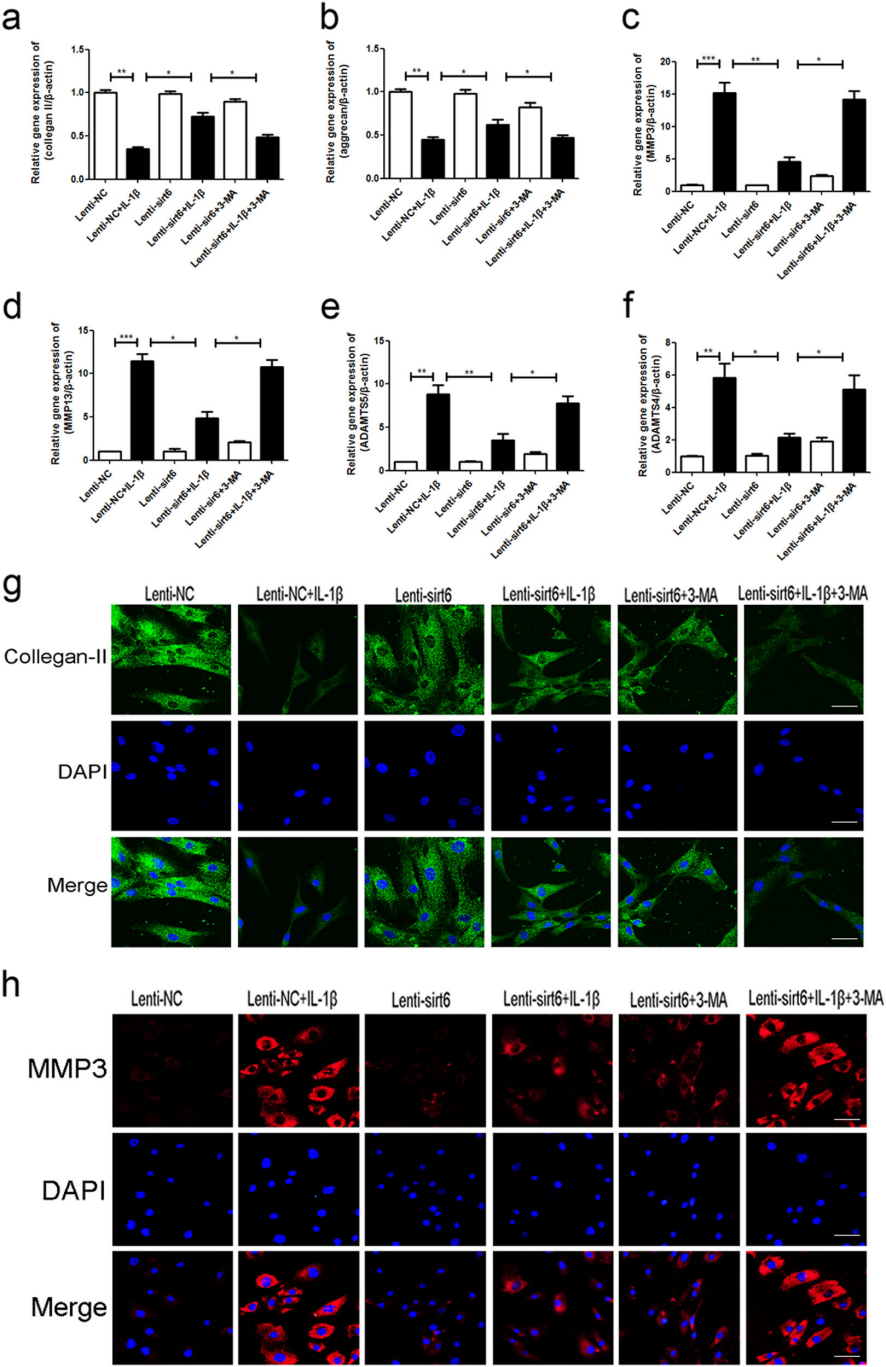
Sirt6 regulated the expression levels of degeneration-associated proteins via autophagy of human NP cells **a**–**f** PCR assay of collegan-II, aggrecan, MMP-3, MMP-13, ADAMT4, and ADAMT5 in NP cells of each group as treated above; columns represent mean ± SD. Significant differences between the treatment and control groups are indicated as *P < 0.05, **P < 0.01, ***P < 0.001, *n* = 5. **g**,** h** Immunofluorescence of collegan-II and MMP-3 in NP cells of each group as treated above (scale bar: 50 μm)

To explore whether autophagy was associated with Sirt6-mediated catabolic processes, we measured the levels of main ECM protein (collagen-II and aggrecan) and main matrix degrading enzymes (MMP3, MMP13, ADAMT4, and ADAMT5) in human NP cells, using PCR and immunofluorescence assay. As shown in Fig. 7a–f, IL-1β greatly reduced the level of messenger RNA (mRNA) encoding collagen II and aggrecan, but increased the level of MMP3, MMP13, ADAMT4, and ADAMT5 mRNA. However, sirt6 attenuated ECM catabolism. These data were consistent with the immunofluorescence results (Fig. 7g and h). Of note, all actions of IL-1β were reversed upon autophagy activation via sirt6 overexpression, as confirmed by addition of the autophagy inhibitor 3-MA.

### Sirt6 overexpression ameliorated puncture-induced IDD in vivo

**Fig. 8:**
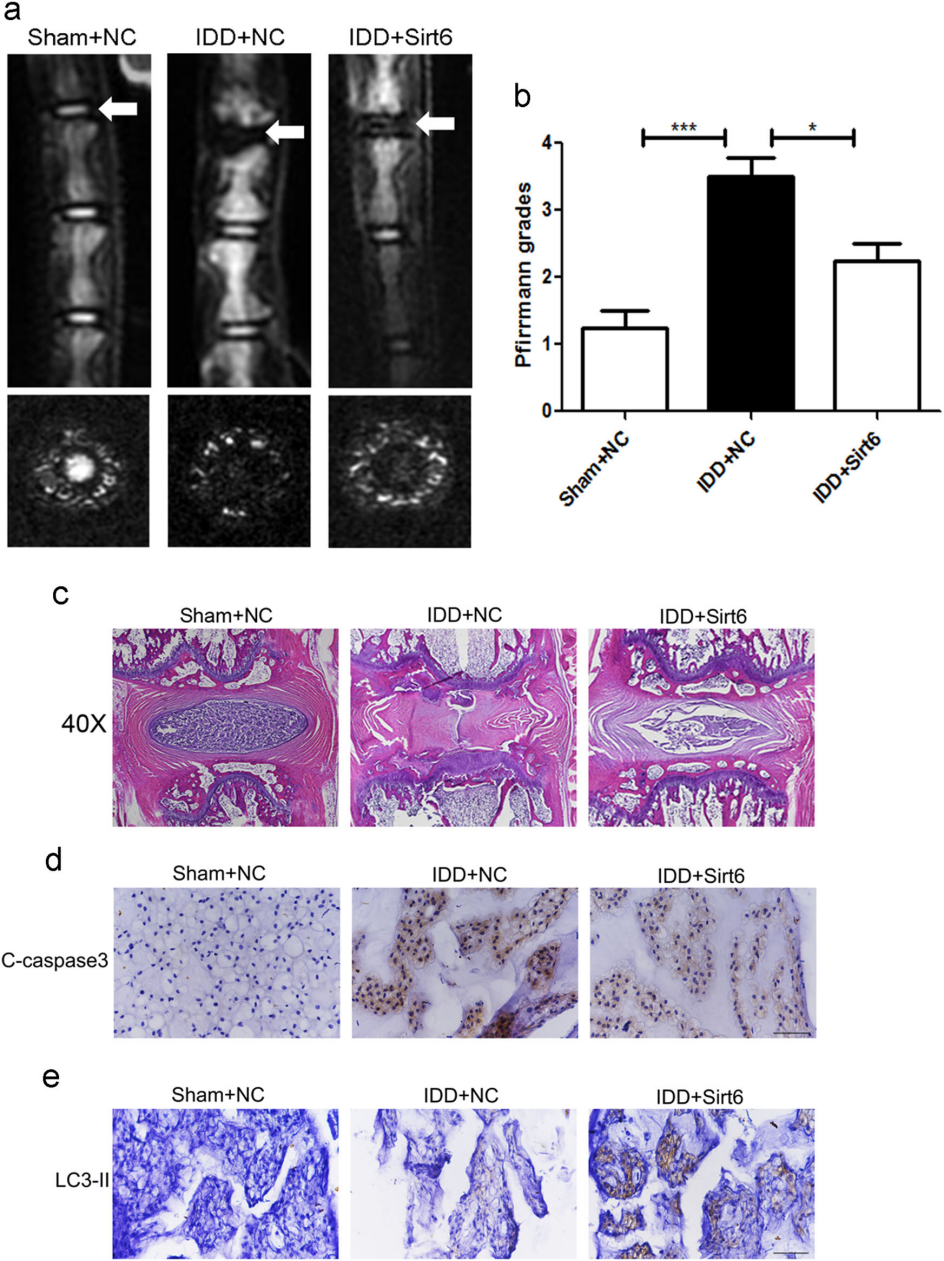
Sirt6 overexpression ameliorated puncture-induced IDD in vivo **a**, **b** T2-weighted MRI and relative Pfirrmann MRI grade scores of a rat tail with a needle-punctured disc at 8 weeks in each group (white arrows); columns represent mean ± SD. Significant differences between the treatment and control groups are indicated as *P < 0.05, ***P < 0.001, *n* = 5. **c** HE staining of each group. **d**, **e** Immunohistochemical staining of cleaved-caspase3 and LC3-II expression in the disc samples of each group (scale bar: 200 μm)

The extent of rat IDD was assessed by MRI and Pfirrmann grading. Figure 8a and b show that, at 8 weeks after puncture, sirt6 transduction was associated with higher T2-weighted signal intensities than those of the control group. The Pfirrmann scores were also significantly lower in rats transfected with sirt6 than controls. The transfection efficiency of Lenti-sirt6 (14 days post transfection) was showed in Supplementary Figure S3. These data were confirmed by H&E staining (Fig. 8c). Compared with the Sham+NC (Lenti-NC) group, the size of the NP in the IDD + NC group was significantly decreased and the fibrous ring was markedly more irregular (clustered), indicating severe NP cell degeneration. Lenti-sirt6 transfection significantly alleviated these degenerative changes (IDD + Sirt6 group). Importantly, immunohistochemical staining showed that sirt6 attenuated the expression of cleaved caspase 3 and activated autophagy in rat disc tissues (Fig. 8d and e), confirming the in vitro data.

## Discussion

Senescence and the associated apoptosis contribute to the pathological changes characteristic of IDD^19^. After chronic prolonged replication, senescence may be a natural feature of disc aging (replicative senescence). However, the process may be accelerated by pathological events including oxidative stress and inflammation (stress-induced premature senescence)^20^. Senescence blocks the cell cycle, reduces cellular viability, and increases the levels of catabolic cytokines and ECM-degrading enzymes^21,22^. During IDD, senescent NP cells secrete ROS and proinflammatory cytokines (tumor necrosis factor (TNF)-α, IL-1β, and IL-6), which in turn accelerate senescence of neighboring cells, increase inflammation, and trigger apoptosis of intervertebral disc cells^22,23^. Chung et al.^24^ found that telomere extension via telomerase transduction attenuated premature senescence of NP cells with recovery of cellular function^24^. Inhibition of NP cell apoptosis by hBMP-7, TGFβ1, sirt1, and several small-molecule drugs such as metformin ameliorated disc degeneration^25–27^. Anti-senescence and anti-apoptosis therapies inhibit IDD.

Sirt6, a member of the sirtuin family of histone deacetylases, exert therapeutic effects in many cellular processes including inflammation, apoptosis, aging, metabolism, and stress-resistance. Sirt6 deficiency triggers cellular senescence and apoptosis^28^. Cardus et al.^29^ found that sirt6 protected endothelial cells from telomere and DNA damage, preventing the onset of premature senescence^29^. Maksin-Matveev et al.^30^ found that sirt6 overexpression protected cardiomyocytes from the onset of necrosis/apoptosis following hypoxia. We previously showed that sirt6 ameliorated osteoarthritis by inhibiting cellular senescence and ECM degeneration^31^. Sirt6 protects NP ECM metabolism by suppressing NF-κB signaling in vitro^32^. However, any role for sirt6 in the regulation of NP cell senescence and apoptosis during IDD has not been previously explored. We found that Sirt6 expression declined both in aging humans and rat NP cells. We used p53, p21, p16, and SA-β-gal levels as canonical markers of cellular senescence. Sirt6 overexpression reduced the levels of these markers in NP cells undergoing replicative senescence; Sirt6 suppressed such senescence.

The levels of proinflammatory cytokines, including IL-1β and TNF-α, increase in aged and degenerative discs of both animals and humans^33,34^. Proinflammatory IL-1β is the most important cytokine in this context, being directly involved in the secretion of many proinflammatory factors such as TNF-α and IL-6, in turn increasing the expression levels of MMPs, disturbing the balance of ECM metabolism, and impairing ECM turnover in intervertebral discs^35^. IL-1β induced both apoptosis and premature senescence of chondrocytes and NP cells^36,37^. Here, we used IL-1β to mimic the pathophysiology of IDD in vitro. IL-1β increased the level of senescence-related proteins and NP cell apoptosis. As expected, sirt6 overexpression reduced the expression of senescence-related proteins and inhibited the SA-β-gal activity induced by IL-1β. Moreover, sirt6 transfection significantly attenuated NP cell apoptosis. Sirt6 attenuated the cellular stress that induces premature senescence and apoptosis.

IL-1β induced apoptosis and autophagy in degenerative human NP cells. Aging degenerative cells exhibited increased autophagy, which is also a feature of other degenerative diseases including osteoarthritis, neurodegeneration, and diabetes^38–40^. Autophagy features catabolism of dysfunctional organelles and proteins to maintain cellular homeostasis and prevent cellular stress^16^. Increasing evidence suggests that autophagy protects cells from inflammation, oxidative stress, and endoplasmic reticulum stress^41^. Autophagy is closely associated with aging and apoptosis. Autophagy inhibition with 3-MA abolished the effects of metformin to protect NP cells against apoptosis and senescence^26^. Autophagy mediated the anti-apoptotic effects of sirt1 induced by nutrient deprivation of human NP cells^42^. Sirt6 attenuated cigarette smoke extract-induced premature senescence of human bronchial epithelial cells by regulating IGF-Akt-mTOR-induced autophagy^43^. He et al.^44^ found that sirt6 played a critical role in protecting against atherosclerosis, decreasing foam cell formation via enhancing autophagy flux. Sirt6 induced autophagy, and sirt6 inhibition attenuated the autophagy of neuronal cells under oxidative stress^45^. We thus hypothesize that the potent anti-apoptotic and anti-senescent effects of sirt6 reflect autophagy activation. We found that sirt6 transfection activated NP cell autophagy. We then showed that the classical autophagy inhibitors, 3-MA and CQ, reversed the therapeutic effects of sirt6. Furthermore, sirt6 overexpression in NP cells markedly reduced the increase in MMP3, MMP13, ADAMT4, and ADAMT5 level induced by IL-1β, enhanced the collagen II and aggrecan expression reduced by IL-1β, which were reversed by both autophagy inhibitors (3-MA and CQ), suggesting that the protective effects of sirt6 were mediated via autophagy. Moreover, we noted that sirt6 overexpression inhibited the mTOR signaling, which played a crucial role in regulating autophagy, indicating sirt6 promoted autophagosome formation via regulation of mTOR signaling in NP cells. In addition, we found, in vivo, that sirt6 transfection enhanced autophagy and suppressed IDD in the annulus needle puncture model. Thus, the protective role played by sirt6 during IDD development is attributable, at least in part, to activation of autophagy in NP cells.

In conclusion, we found that sirt6 played a critical role in IDD development by attenuating senescence, including replicative senescence and stress-induced premature senescence, and inhibits apoptosis in NP cells by regulating mTOR/autophagy in a model of IDD. Sirt6 may be a potential target for future therapeutic interventions seeking to attenuate IDD.

## Methods and materials

### Reagents and antibodies

Recombinant human IL-1β was obtained from Peprotech (Rocky Hill, NJ, USA). Antibodies against cleaved caspase 3, LC3, Beclin-1, and p62 were purchased from Cell Signaling Technology (Beverly, MA, USA). Antibodies against Bax, Bcl-2, matrix metalloproteinase-3 (MMP-3), p21, p-p53 and p53 were purchased from Santa Cruz Biotechnology (Santa Cruz, CA, USA). Antibodies against sirt6, P16 and collagen-II were the products of Abcam (Cambridge, MA, USA). Other reagents were obtained from Sigma (St. Louis, MO, USA) unless noted otherwise.

### NP cells culture

All human disc tissues were obtained from patients undergoing elective spinal surgery. The work was given official approval by the Ethics Committee of the Second Affiliated Hospital of Wenzhou Medical University. We obtained written informed consent from patients or relatives prior to tissue collection. Human and rat NP cells were isolated as described previously (Supplementary Figure S4)^46^. The cells were added to six-well plates at 1 × 10^5^ cells per well. After pretreatment (or not) with Lenti-sirt6 or Lenti-NC (control) viruses, the cells were grown with or without addition of IL-1β (10 ng/ml) and autophagy inhibitor 3-methyladenine (3-MA, 10 μM) or chloroquine (CQ, 100 μM) for 24 h, at which time the cells were 70–80% confluent. The cells were then harvested.

### Lentivirus transfection

Sirt6 was overexpressed via transfection of Lenti-sirt6 (Invitrogen, Carlsbad, CA, USA). The cells were transfected with Lenti-sirt6 or Lenti-NC at a confluence of 30–50%; >95% of the cells were viable 12 h later. The medium was then changed, the cells incubated for a further 3 days, and passaged. Transfection efficacies were measured via western blotting.

### Senescence analysis

The level of senescence was measured by senescence-associated β-galactosidase (SA-β-gal) staining kit (Beyotime, Shanghai, China) according to the instruction. Aging NP cells showing higher SA-β-gal activity were stained blue.

### Western blot analysis

The proteins of NP cells were extracted using radioimmunoprecipitation buffer containing protease and phosphatase inhibitors and the protein concentrations were measured by the bicinchoninic acid method (Thermo Fisher Scientific, Rockford, IL, USA). Total proteins were separated via 8–12% (w/v) sodium dodecyl sulfate polyacrylamide gel electrophoresis and blotted onto polyvinylidene fluoride membranes (Bio-Rad, Hercules, CA, USA). After blocking with 5% bovine serum albumin (BSA), the bands were subsequently incubated with primary antibodies (cleaved caspase 3, p-mTOR, mTOR, LC3, Beclin-1, p62, Bax, Bcl-2, sirt6, P16, p-p53, p53, p21, and β-actin), followed by addition of the appropriate secondary antibodies. Bands were visualized and analyzed using the ChemiDicTM XRS+ Imaging System and the Image Lab 3.0 software (Bio-Rad).

### Immunofluorescence staining

At 8 weeks after surgery, the disc tissues were embedded in paraffin. Sections (5 μm thick) were cut, deparaffinized in xylene, and rehydrated in ethanol. And cells cultured on microscopic glasses and treated as described above. After fixation in 4% (v/v) paraformaldehyde, NP cells or tissue sections incubated in 1% (v/v) Triton X-100 for 10 min, blocked with 5% BSA for 30 min, and incubated with primary antibodies against sirt6, LC3, cleaved caspase 3, and collagen II at 4 °C overnight. Appropriate secondary antibodies were added for 1 h, followed by 4′,6-diamidino-2-phenylindole (DAPI) staining (7 min). Images were captured with the aid of a Nikon ECLIPSE Ti microscope (Nikon, Japan).

### Apoptosis analysis

The terminal deoxynucleotidyl transferase (TdT) dUTP nick end labeling (TUNEL) staining was used to measure apoptosis. After fixation in 4% (v/v) paraformaldehyde for 1 h, and incubation with 3% (v/v) H_2_O_2_ and 0.1% (v/v) Triton X-100 for 10 min, the cells were stained with a reagent of the In Situ Cell Death Detection Kit (Roche Molecular Biochemicals, Biospace, USA) and DAPI, and microscopically observed. Caspase-3 activity was measured using a Caspase-3 Activity Assay Kit (Cell Signaling Technology, Beverly, USA).

### RT-PCR

The total RNA of cells was extracted using the TRIzol method. The complementary DNA was synthetized and then amplificated using the PrimeScript-RT reagent kit and SYBR Premix Ex Taq (Sangon). The level of target gene was analyzed using the DDCt method, as described^47^.

### Transmission electron microscopy

After fixation in 2.5% (w/v) glutaraldehyde overnight and post-fixed in 2% (w/v) osmium tetroxide, the NP cells were stained with 2% (w/v) uranyl acetate, and dehydrated in acetone. Semi-thin sectioning and toluidine blue staining followed. Images were captured with the aid of a Hitachi transmission electron microscope.

### Annulus needle puncture and drug treatment

Adult male Sprague Dawley rats (200–220 g; Animal Center of Chinese Academy of Sciences, Shanghai, China) were anaesthetized via injection of 10% (w/v) chloral hydrate (3.6 mL/kg, ip) and randomly divided into three groups: SHAM + Lenti-NC, IDD + Lenti-SIRT6, and IDD + Lenti-NC. Using aseptic techniques, a small sagittal skin incision was performed to expose the Co7/8 disc, which was then punctured with a 30-gauge syringe needle as previously described^48^. The needle was inserted into the NP of tail (from the dorsal side to ventral side), parallel to the endplate. Before extraction, the needle was allowed to penetrate either the entire disc or the disc to a depth of 5 mm, was rotated through 360°, and then held in position for 30 s. Next, the Lenti-NC or Lenti-SIRT6 construct was injected into the disc. The SHAM group received an injection of Lenti-NC only. The rats were then returned to their cages. All operators were blinded to mouse grouping.

### Immunohistochemical examination

All discs were embedded in paraffin and cut into sections (5 μm). The sections were deparaffinised in xylene, rehydrated in ethanol, and blocked by addition of 3% (v/v) H_2_O_2_ for 10 min followed by incubation in 5% BSA for 30 min. After incubation with primary antibodies (anti-LC3, anti-cleaved caspase 3), the sections were incubated with the respective second antibodies and counterstained with hematoxylin. Images were captured using a light microscope.

### Hematoxylin-eosin staining

To measure the extent of IDD after surgery, all rats were killed 8 weeks after surgery and disc tissues sections (5 µm) were cut and treated as described above for hematoxylin-eosin (HE) staining. Images were captured using a light microscope.

### Magnetic resonance imaging

Magnetic resonance imaging (MRI) of the coccyx was performed 8 weeks after surgery. All rats were anesthetised throughout the examinations, and their tails straightened. We subjected five rats of each group (total, 15) to sagittal and horizontal T2-weighted imaging with a 3.0-T clinical magnet (Philips Intera Achieva 3.0MR). T2-weighted sections were set as followed: a fast-spin echo sequence with a time-to-repetition of 5400 ms and a time-to-echo of 920 ms; a 320 (h) × 256 (v) matrix; a field of view of 260°; and four excitations. The section thickness was 2 mm and the gap 0 mm. All MR images were analyzed in a blinded manner using the IDD classification of Pfirrmann et al.^49^

### Data analysis

All data are the means ± standard deviations (SDs). Results were compared via Graphpad Prism (USA) (one-way analysis of variance and Tukey’s post hoc test). *P* values <0.05 were considered statistically significant.

## Electronic supplementary material

Supplementary Figure S1

Supplementary Figure S2

Supplementary Figure S3

Supplementary Figure S4

Supplementary Figure Legends
