# Supplementary figures and images for: Sirt6 overexpression suppresses senescence and apoptosis of nucleus pulposus cells by inducing autophagy in a model of intervertebral disc degeneration
Source: Cell Death Dis. 2018 Jan 19;9(2):56. doi: 10.1038/s41419-017-0085-5 (PMC5833741; doi:10.1038/s41419-017-0085-5)

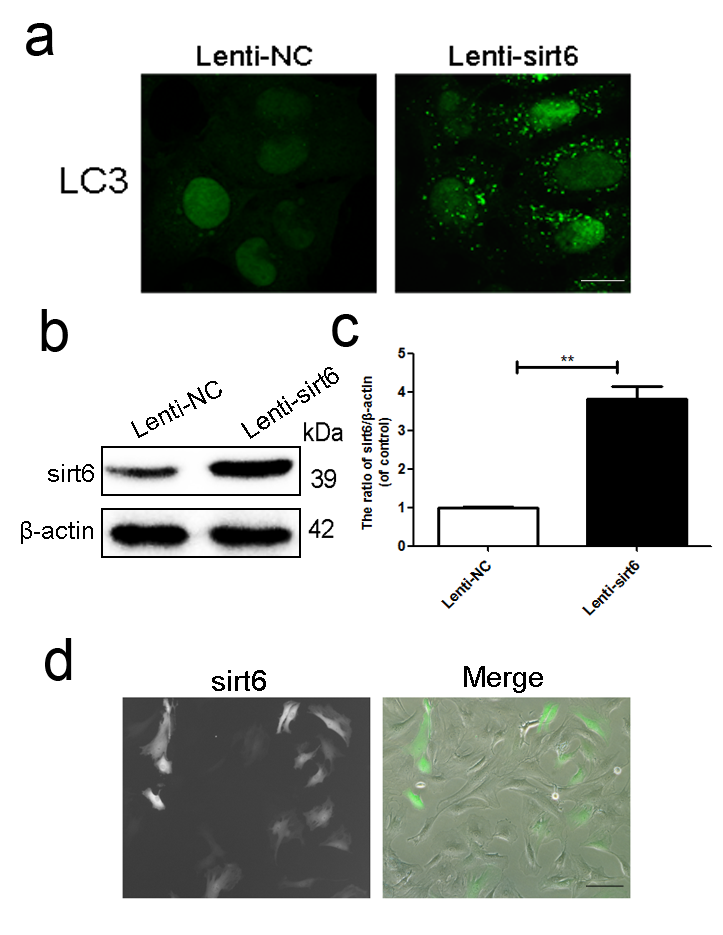

Supplement: Supplementary file 1 — Supplementary Figure S1 [file 41419_2017_85_MOESM1_ESM.tif]

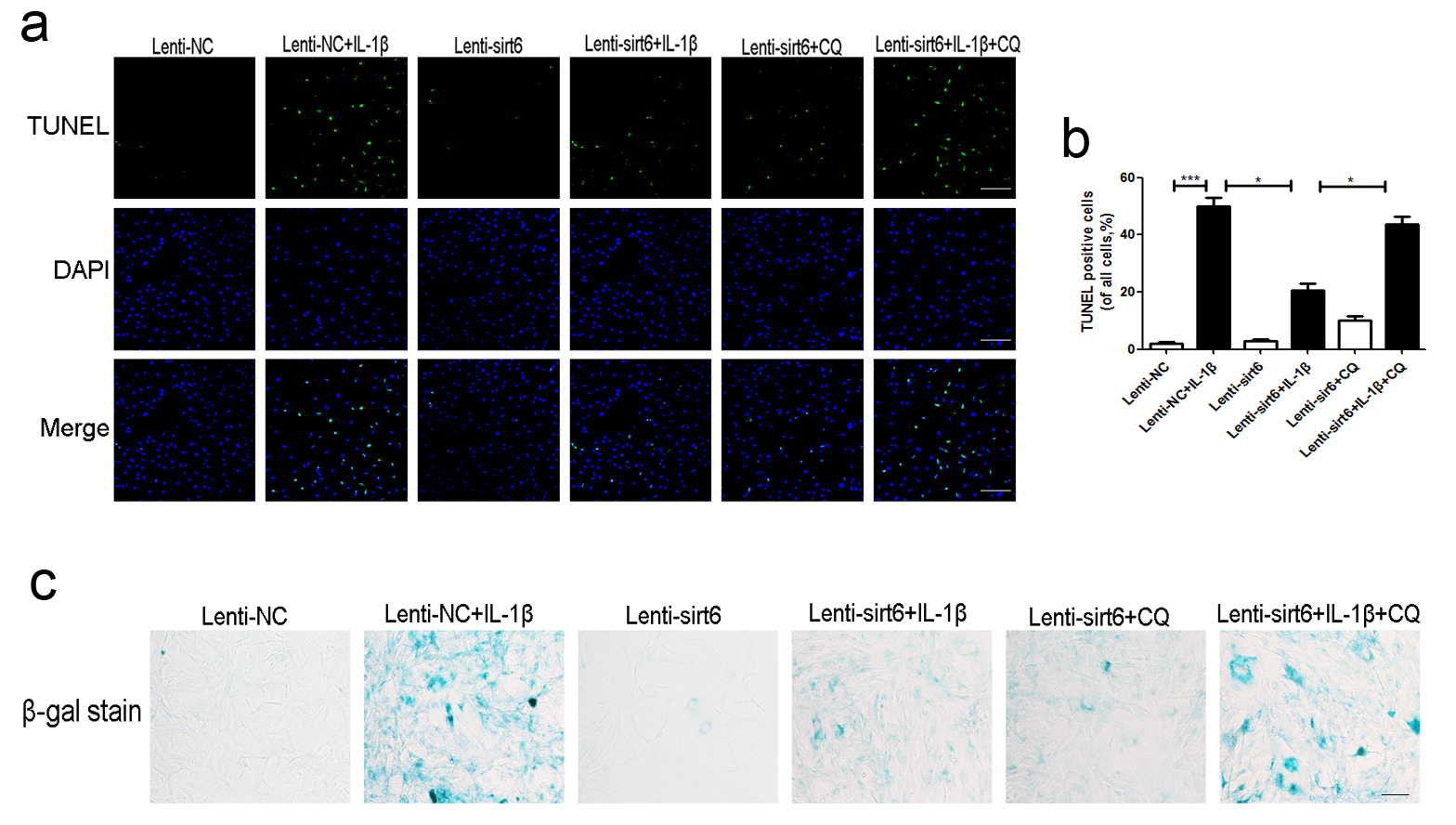

Supplement: Supplementary file 2 — Supplementary Figure S2 [file 41419_2017_85_MOESM2_ESM.tif]

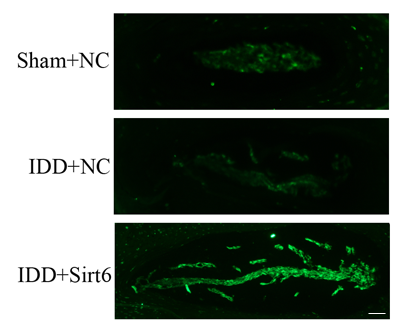

Supplement: Supplementary file 3 — Supplementary Figure S3 [file 41419_2017_85_MOESM3_ESM.tif]

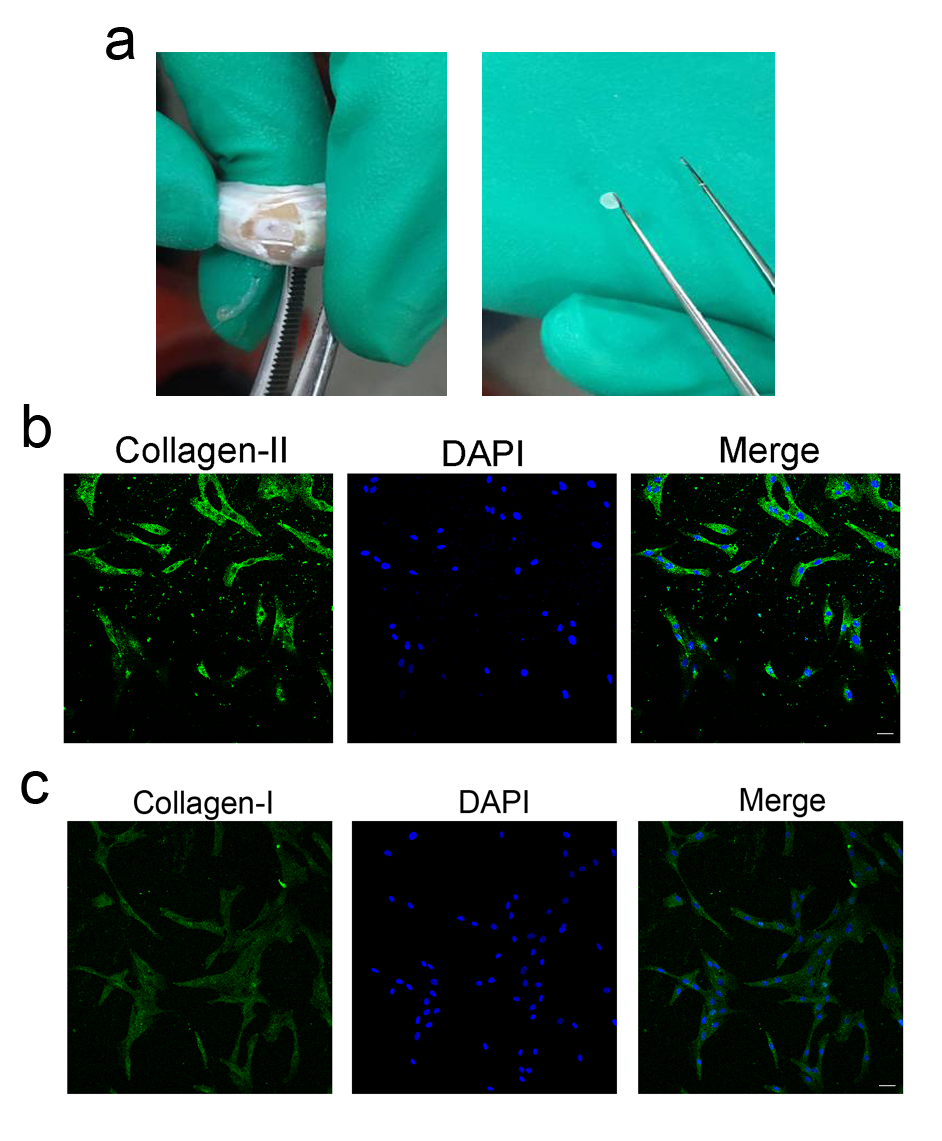

Supplement: Supplementary file 4 — Supplementary Figure S4 [file 41419_2017_85_MOESM4_ESM.tif]
