# Supplementary material for: Sirt6 overexpression suppresses senescence and apoptosis of nucleus pulposus cells by inducing autophagy in a model of intervertebral disc degeneration
Source: Cell Death Dis. 2018 Jan 19;9(2):56. doi: 10.1038/s41419-017-0085-5 (PMC5833741; doi:10.1038/s41419-017-0085-5)
Supplement: Supplementary file 5 — Supplementary Figure Legends [file 41419_2017_85_MOESM5_ESM.docx]

**Supplementary Figure Legends**

**Supplementary Figure S1** Sirt6 transfection activated autophagy in human NP cell. (a) Immunofluorescence of LC3 in NP cells of each group as treated above (scale bar: 10μm). (b-c) Representative western blots and quantification data of sirt6 protein in NP cells of each group; columns represent mean ± SD, Significant differences between the treatment and control groups are indicated as **P<0.01, n=5. (d) Cell immunofluorescence of sirt6 in NP cells treated by low titer of Lenti-sirt6 (scale bar: 50μm).

**Supplementary Figure S2** Inhibition of autophagy via activating mTOR signaling with CQ attenuated the anti-apoptosis and anti-senescence effects of sirt6 in human NP cell. (a-b) TUNEL assay was performed to measure the apoptotic level in NP cells of each group as treated above (scale bar: 50μm). (c) SA-β-gal staining assay was performed in NP cells of each group as treated above (scale bar: 50μm).

**Supplementary Figure S3** Transfection efficiency of Lenti-sirt6 in tissue slice (14 days post transfection). Immunofluorescence of sirt6 in tissue slice (scale bar: 200μm).

**Supplementary Figure S4** Extraction steps and identification methods of NP cells. (a)The gel-like NP of rat was separated from the AF using an ophthalmic tweezers. (b-c) Cell fluorescence results of collegan-I and collegan-II in cells (scale bar: 50μm).
